# Supplementary material for: REPTOR and CREBRF encode key regulators of muscle energy metabolism
Source: Nat Commun. 2023 Aug 15;14:4943. doi: 10.1038/s41467-023-40595-1 (PMC10427696; doi:10.1038/s41467-023-40595-1)
Supplement: Supplementary file 1 — Supplementary Information [file 41467_2023_40595_MOESM1_ESM.pdf]

SUPPLEMENTARY INFORMATION FILE

Supplementary Figures and Figure legends

Supplementary Table 1 with qRT-PCR primers used in this work

Source Data for Supplementary Figure 2a

Source Data for Supplementary Figure 5e

Source Data for Supplementary Figure 10i

Supplementary references

Supplementary Figure 1

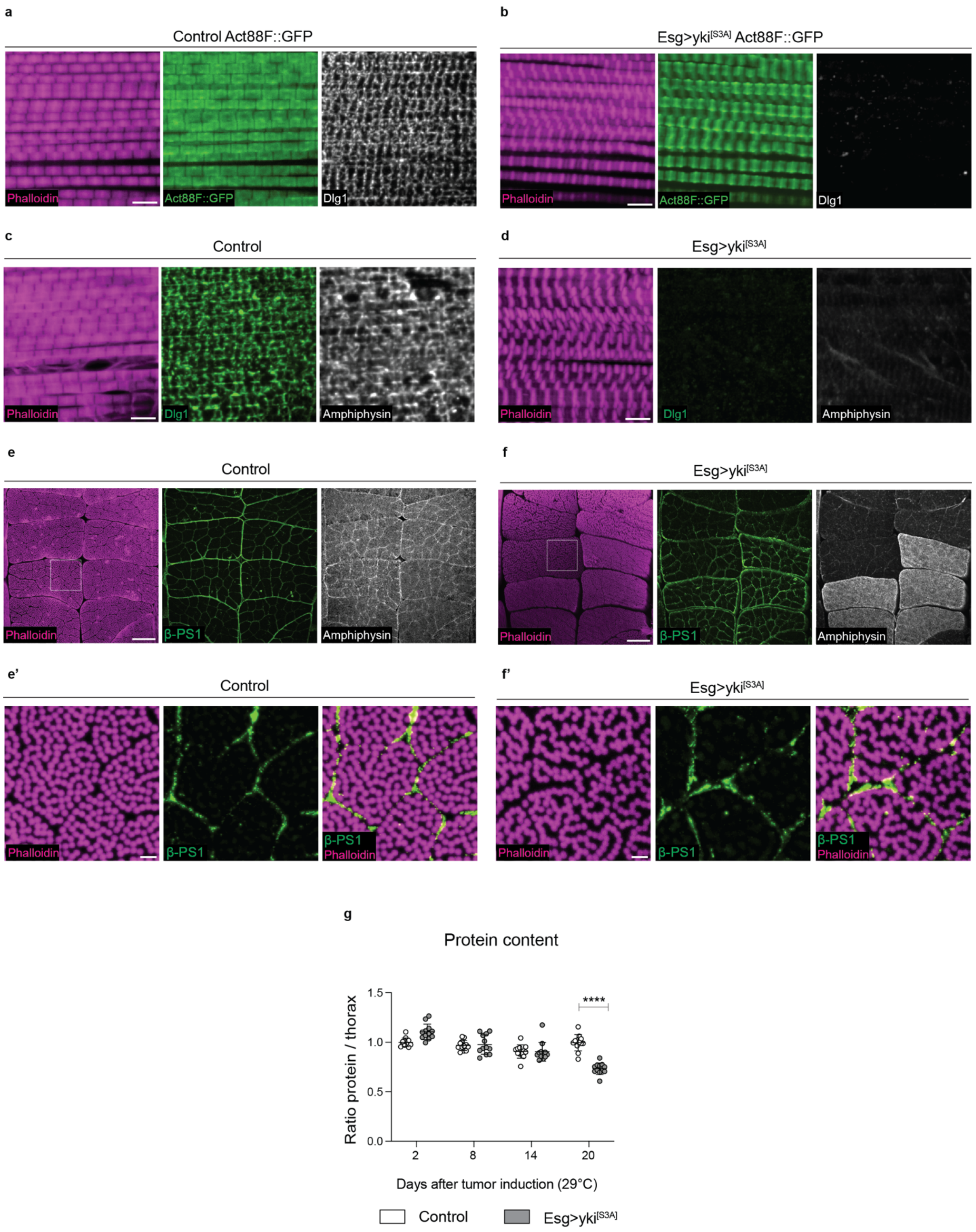

Supplementary Figure 1. Characterization of the myofiber degradation phenotype in *Esg>yki*$^{[S3A]}$ thoraces. a-f Immunostaining of flight muscles of control (**a, c, e**) or *Esg>yki*$^{[S3A]}$ thoraces (**b, d, f**). All panels show myofibrils labelled with phalloidin (magenta). Samples expressing Actin88F::GFP (green) were stained with anti-GFP to label Actin88F in the sarcomeres (**a, b**). The t-tubule network was labelled with Dlg1 (gray in **a, b** and green in **c, d**), or Amphiphysin (gray in **c-f**). In cross-sections of thoraces, β-PS1 (green) was used to mark the plasma membrane of flight muscles **(e, f).** White boxes indicate zoomed in areas shown in **e'** and **f'**. **g** Protein content in thoraces after several days of tumor induction (p<0.0001****). Protein content was calculated from the same samples used in Fig. 1b, c. N=12 biologically independent samples, N=8 thoraces per sample. Results were reproduced in three independent experiments.

Data shows mean with ± SD (g). Values were normalized to the mean of control samples of 2 days after tumor induction (**g**). Statistical analysis was done using two-way ANOVA with Sidak correction test for multiple comparisons. Scale bar is 5 µm (**a-d**), 50 µm (**e, f**) or 10 µm (**e', f'**). Source data are provided as a Source Data file.

Supplementary Figure 2

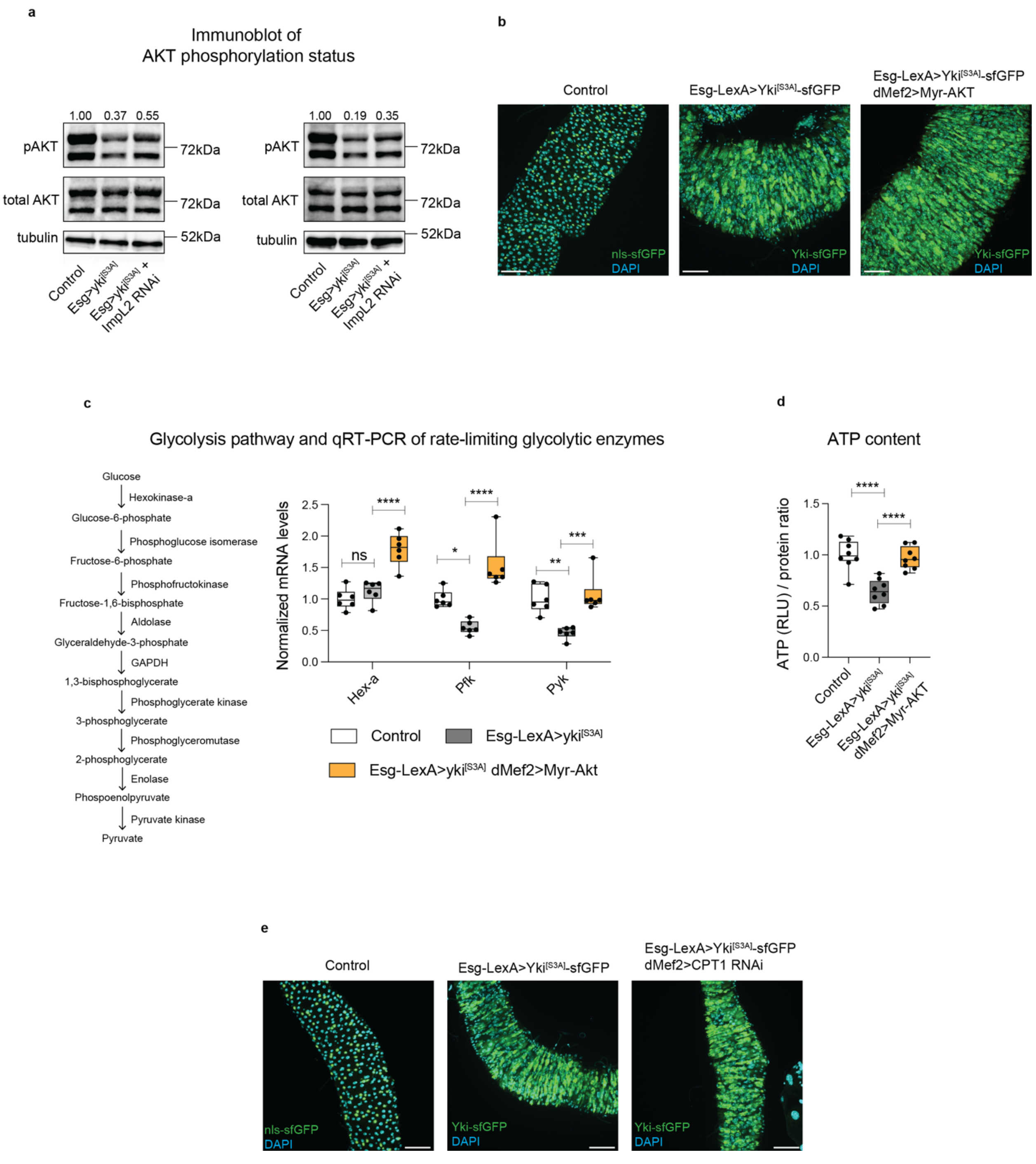

Supplementary Figure 2: Characterization of the effect of elevating insulin signaling or knocking down *CPT1* in muscle of *Esg>yki$^{[S3A]}$* flies. **a** Protein levels of phosphorylated AKT (pAKT) and total AKT in thoraces. N=2 biologically independent samples. Results were reproduced in three independent experiments. Numbers indicate densitometry of bands normalized to control samples. **b** Immunostaining of posterior midguts expressing nuclear superfolder GFP (nls-sfGFP) or *yki$^{[S3A]}$*-sfGFP with anti-GFP (green) and DAPI (cyan). **c** mRNA levels in thoraces of the glycolytic rate-limiting enzymes *Hexokinase-a* (*hex-a), Phosphofructokinase* (*pfk*) and *Pyruvate Kinase* (*pyk*) (*hex-a*: Ctrl vs *yki$^{[S3A]}$* p=0.547 ns, *yki$^{[S3A]}$* vs *yki$^{[S3A]}$ Myr-AKT* p<0.0001****. *pfk:* Ctrl vs *yki$^{[S3A]}$* p=0.012*, *yki$^{[S3A]}$* vs *yki$^{[S3A]}$ Myr-AKT* p<0.0001****; *pyk:* Ctrl vs *yki$^{[S3A]}$* p=0.0012**, *yki$^{[S3A]}$* vs *yki$^{[S3A]}$ Myr-AKT* p=0.0004***). N=6 biologically independent samples, N=5-10 thoraces per sample. **d** ATP content in thoraces (Ctrl vs *yki$^{[S3A]}$* p<0.0001****, *yki$^{[S3A]}$* vs *yki$^{[S3A]}$ Myr-AKT* p=0.0001***). N=8 biologically independent samples, N=4 thoraces per sample. Results were reproduced in three independent experiments. **e** Immunostaining of posterior midguts expressing nuclear superfolderGFP (nls-sfGFP) or *yki$^{[S3A]}$*-sfGFP after 12 days of tumor induction with anti-GFP (green) and DAPI (cyan). Samples were analyzed 14 days (**a**), 12 days (**b, c, e**), or 16 days after tumor induction (**d**).

Data shows boxplots (median and quartiles) with whiskers (minimum to maximum) (**c, d**). Values were normalized to the mean of control samples (**c, d**). Statistical analysis was done by one-way ANOVA with Sidak correction test for multiple comparisons (**c, d**). Scale bar is 50 µm in **b, e.** Source data are provided as a Source Data file.

Supplementary Figure 3

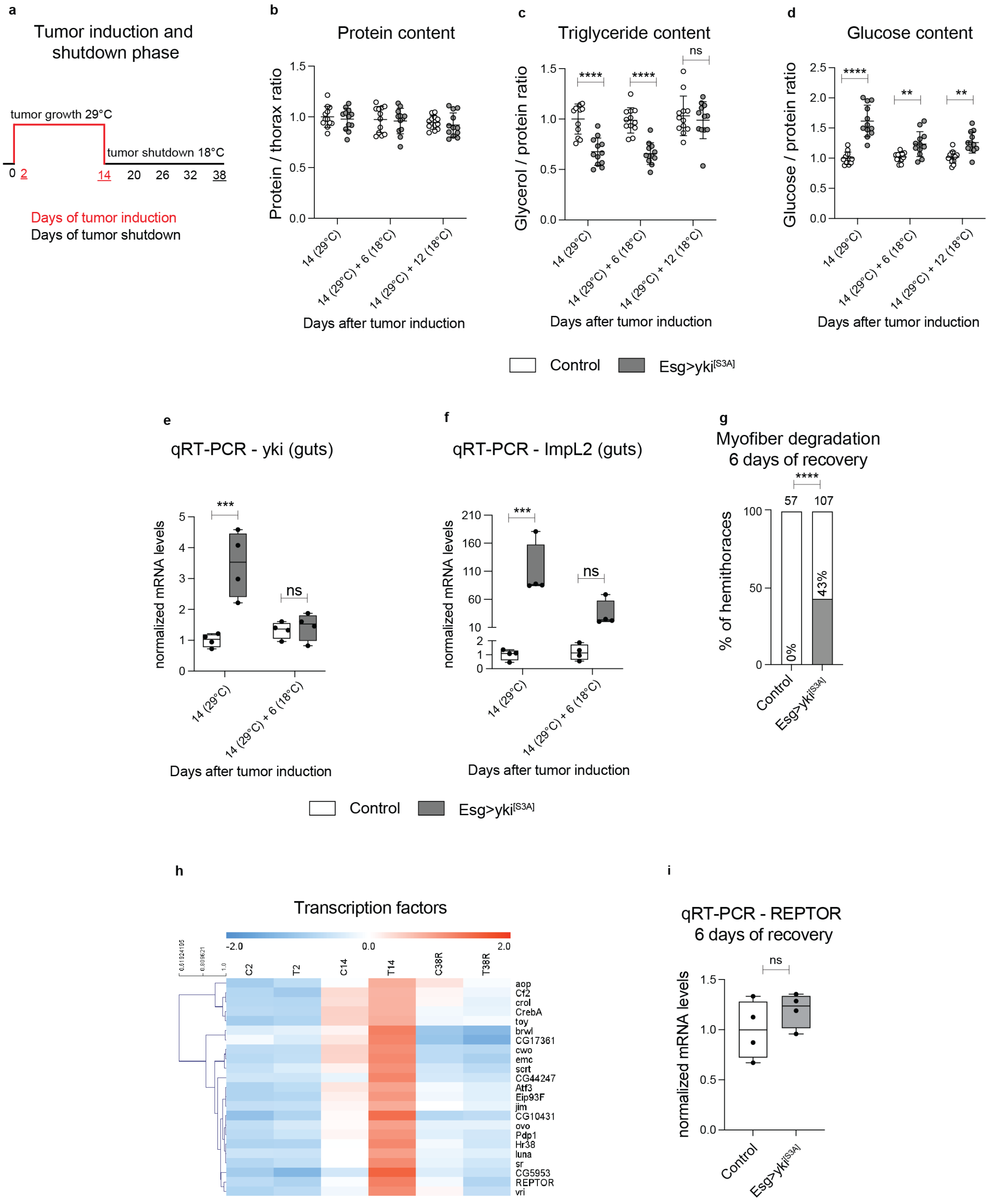

Supplementary Figure 3. Time-course RNA-seq in *Esg>yki$^{[S3A]}$* thorаces. a Model showing the duration of the tumor induction (red line - 29°C) and the tumor shutdown phase (black line - 18°C). Underlined days were used as time points for RNA-seq analysis of thoraces: day 2 and 14 of tumor induction at 29°C, and day 38 that spans a recovery phase of 24 days at 18°C after tumor shutdown. **b-d** Protein (**b**), triglyceride (**c**) and glucose (**d**) content from same thoraces during the recovery phase after 14 days of tumor induction (Triglycerides: 14 days p<0.0001****, 14+6 days p<0.0001****, 14+12 days p=0.87 ns. Glucose: 14 days p<0.0001****, 14+6 days p=0.0076**, 14+12 days p=0.0019**). N=12 biologically independent samples, N=8 thoraces per sample. Results were reproduced in three independent experiments. **e, f** mRNA levels of *yki* (**e**) and *ImpL2* (**f**) in guts (*yki*: 14 days p=0.0092***, 14+6 days p=0.96 ns. *ImpL2*: 14 days p=0.0002***, 14+6 days p=0.20 ns). N=4 biologically independent samples, N=6-10 guts per sample. **g** Percentage of hemi-thoraces showing signs of myofiber degradation after 14 days of tumor induction and 6 days of recovery (p<0.0001****). Total number of hemi-thoraces scored is shown for each genotype. **h** Heat map showing the transcription factors with reverse pattern of expression for control (C) and *Esg>yki$^{[S3A]}$* (T) thoraces during tumor induction (C2 to C14; T2 to T14) and recovery phase (C14 to C38R; T14 to T38R). **i** mRNA levels of *REPTOR* in thoraces after 6 days of recovery (14 days at 29°C + 6 days at 18°C, p=0.30 ns) normalized to *Rp49*. N=4 biologically independent samples, N=5-10 thoraces per sample.

Data shows mean with ± SD (**b-d**) or boxplots (median and quartiles) with whiskers (minimum to maximum) (**e, f, i**). Values were normalized to the mean of control samples of 14 days after tumor induction (**b-f**) or control samples (**i**). Statistical analysis was done by two-way ANOVA (**b-f**) with Sidak multiple comparison correction, or using two-tailed *t*-test with Welch's correction (**i**), or two-tailed Fisher's exact test with a confidence interval of 95% for pairwise comparisons between two groups (**g**). Source data are provided as a Source Data file.

Supplementary Figure 4

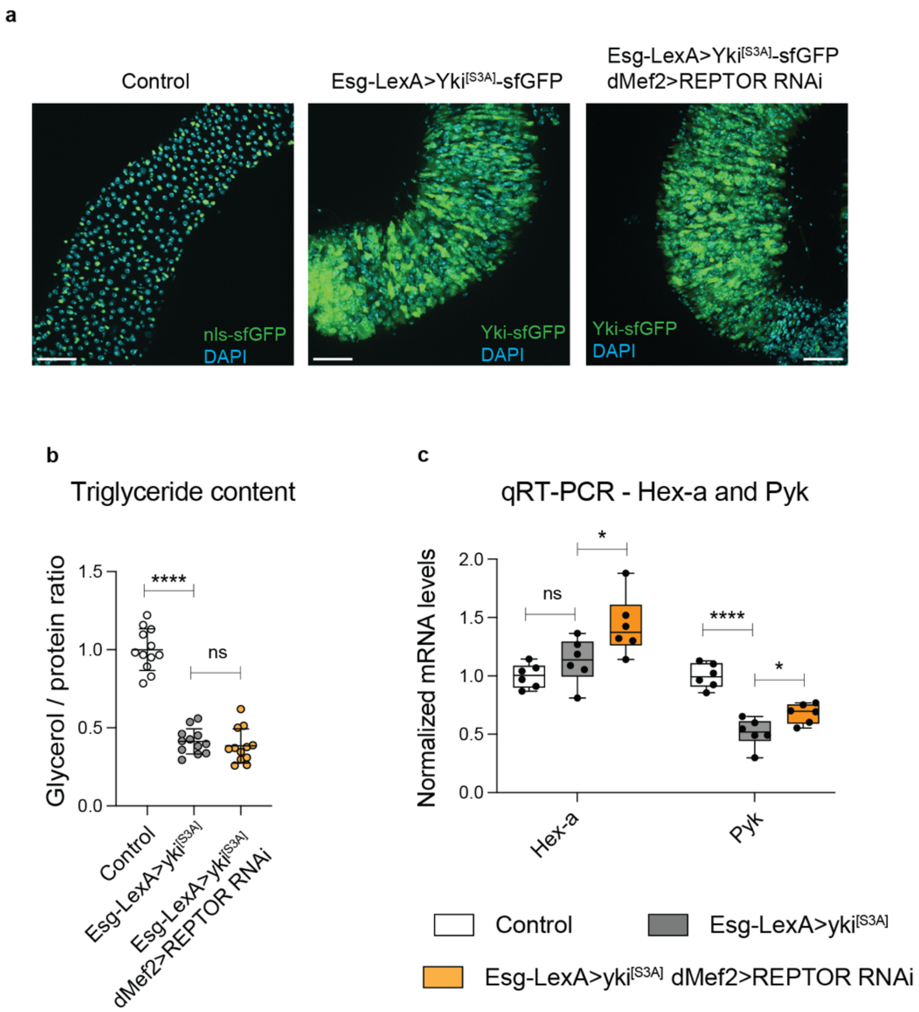

Supplementary Figure 4. Characterization of the effect of *REPTOR* knockdown in muscle of *Esg>yki$^{[S3A]}$* flies. a Immunostaining of posterior midguts expressing nuclear superfolder GFP (nls-sfGFP) or *yki$^{[S3A]}$*-sfGFP with anti-GFP (green) and DAPI (cyan). **b** Triglyceride content in thoraces (Ctrl vs *yki$^{[S3A]}$* p<0.0001****, *yki$^{[S3A]}$* vs *yki$^{[S3A]}$ REPTOR RNAi* p=0.78 ns). N=12 biologically independent samples, N=8 thoraces per sample. Results were reproduced in three independent experiments. **c** mRNA levels in thoraces of the glycolytic rate-limiting enzymes *Hexokinase-a* (*hex-a)* and *Pyruvate Kinase* (*pyk*) (*hex-a*: Ctrl vs *yki$^{[S3A]}$* p=0.46 ns, *yki$^{[S3A]}$* vs *yki$^{[S3A]}$ REPTOR RNAi p*<0.0327*. *pyk:* Ctrl vs *yki$^{[S3A]}$* p<0.0001****, *yki$^{[S3A]}$* vs *yki$^{[S3A]}$ REPTOR RNAi* p=0.0314*). N=6 biologically independent samples, N=5-10 thoraces per sample. Samples were analyzed 12 days after tumor induction (**a-c**).

Data shows mean with ± SD (**b**) or boxplots (median and quartiles) with whiskers (minimum to maximum) (**c**). Values were normalized to the mean of control samples (**b, c**). Statistical analysis was done by using one-way ANOVA with Sidak correction test for multiple comparisons (**b, c**). Scale bar is 50 µm in **a.** Source data are provided as a Source Data file.

Supplementary Figure 5

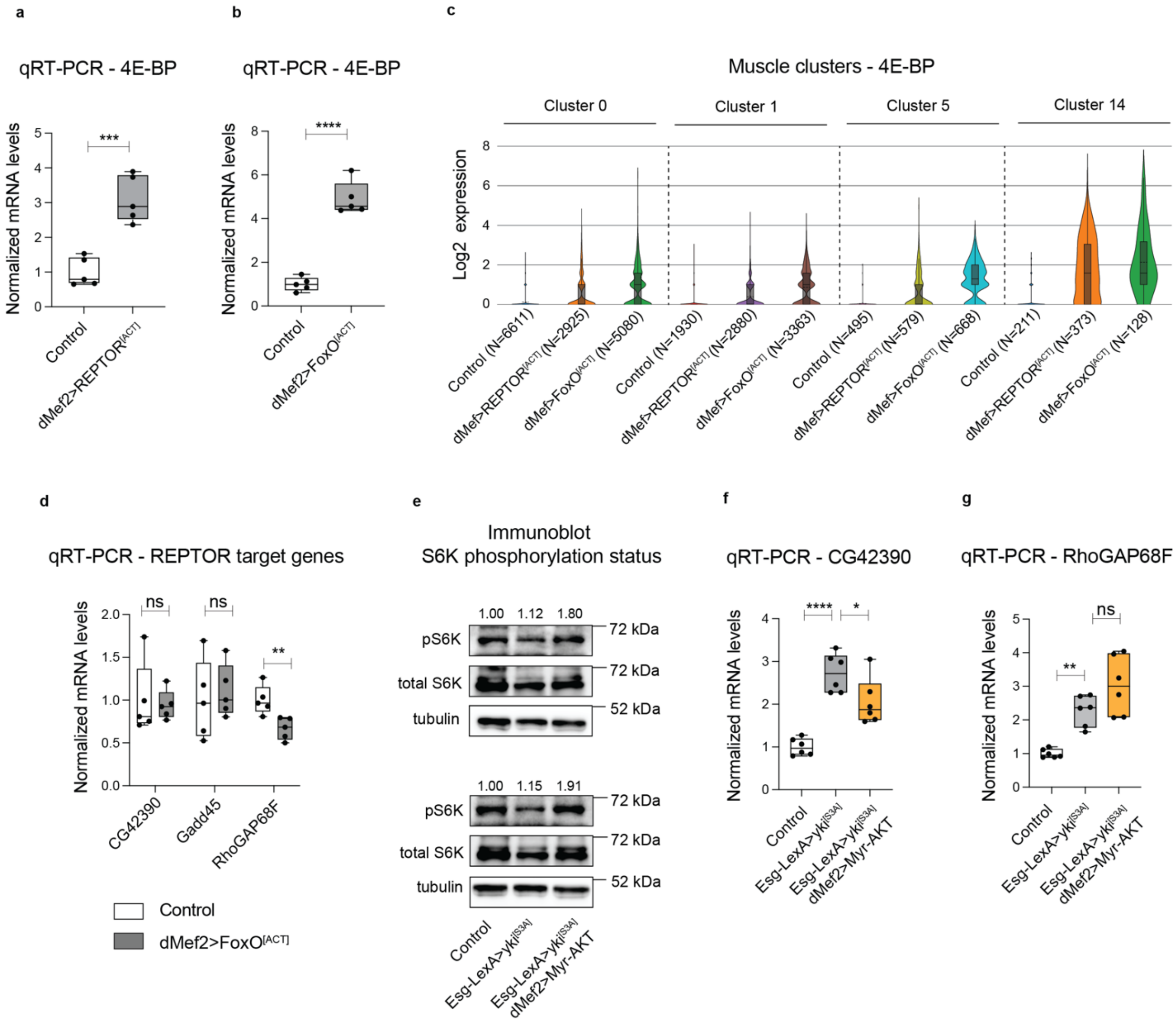

Supplementary Figure 5: Validation of REPTOR target genes in muscle tissue. a, b mRNA levels of *4E-BP* in thoraces upon muscle-specific overexpression of: *REPTOR[ACT]* (p=0.0007***) (**a**), or *FoxO[ACT]* (p<0.0001****) (**b**). N=5 biologically independent samples, N=5-6 thoraces per sample. **c** Violin plots showing expression of *4E-BP* (log2) in nuclei for each muscle cluster. Total number of nuclei analyzed per condition and per cluster is indicated. **d** mRNA levels in thoraces of potential target genes of REPTOR (see Fig. 3d) upon *FoxO[ACT]* induction in muscle (*CG42390* p=0.79 ns, *Gadd45* p=0.69 ns, *RhoGAP68F* p=0.0093**). N=5 biologically independent samples, 5-6 thoraces per sample. **e** Protein levels of p-S6K and total S6K in thoraces. N=2 biologically independent samples. Results were reproduced in three independent experiments. Numbers indicate densitometry of bands normalized to control samples. The p-S6K/S6K ratio between control and *Esg>yki[S3A]* thoraces is similar due to a decrease in total S6K levels, but it is higher when *Myr-AKT* is overexpressed in muscle of *Esg>yki[S3A]* flies. **f, g** mRNA levels in thoraces of *CG42390* (Ctrl vs *yki[S3A]* p<0.0001****, *yki[S3A]* vs *yki[S3A] Myr-AKT* p=0.0301*) (**f**), or *RhoGAP68F* (Ctrl vs *yki[S3A]* p=0.0033**, *yki[S3A]* vs *yki[S3A] Myr-AKT* p=0.07 ns) (**g**) upon elevation of insulin signaling (*Myr-AKT*) in muscle of *Esg>yki[S3A]* flies for 12 days. N=6 biologically independent samples, N=5-10 thoraces per sample.

Data shows boxplots (median and quartiles) with whiskers (minimum to maximum) (**a, b, d, f, g)** or violin plots (median, quartiles and interquartile) **(c)**. Values were normalized to the mean of control samples (**a, b, d, f, g**). Statistical analysis was done by using two-tailed *t*-test with Welch's correction (**a, b, d**) or one-way ANOVA with Sidak correction test for multiple comparisons (**f, g**). Source data are provided as a Source Data file.

Supplementary Figure 6

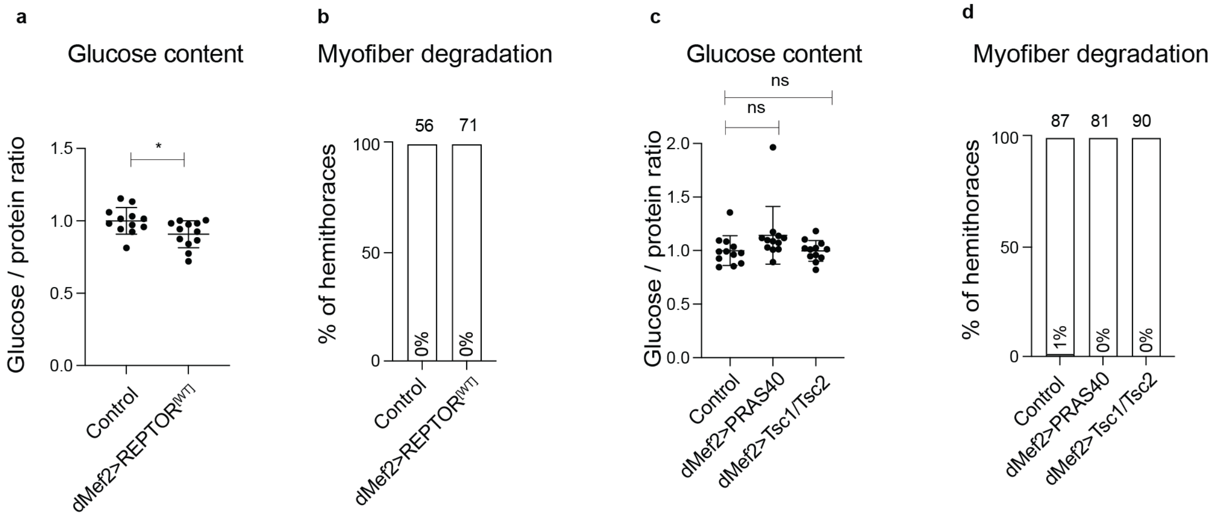

Supplementary Figure 6: Overexpression of *REPTOR[WT]*, *PRAS40* or *Tsc1/Tsc2* in wildtype muscle does not increase glucose content or promote myofiber degradation. a Glucose content in thoraces (p=0.0232*). N=12 biologically independent samples, N=8 thoraces per sample. Results were reproduced in three independent experiments. **b** Percentage of hemi-thoraces showing myofiber degradation. Total number of hemi-thoraces scored for each genotype is shown. *REPTOR[WT]* expression was induced in muscle for 20 days. **c** Glucose content in thoraces (*PRAS40* p=0.13 ns, *Tsc1/Tsc2* p=0.99 ns). N=12 biologically independent samples, N=8 thoraces per sample. Results were reproduced in three independent experiments. **d** Percentage of hemi-thoraces showing myofiber degradation. Total number of hemi-thoraces scored for each genotype is shown. Gene expression was induced for 20 days (**c, d**).

Data shows mean with ± SD (**a, c**). Values were normalized to the mean of control samples (**a, c**). Statistical analysis was done by using two-tailed *t*-test with Welch's correction (**a**), one-way ANOVA with Sidak correction test for multiple comparisons (**c**), or two-tailed Fisher's exact test with a confidence interval of 95% for pairwise comparisons between two groups (**b, d**). Source data are provided as a Source Data file.

Supplementary Figure 7

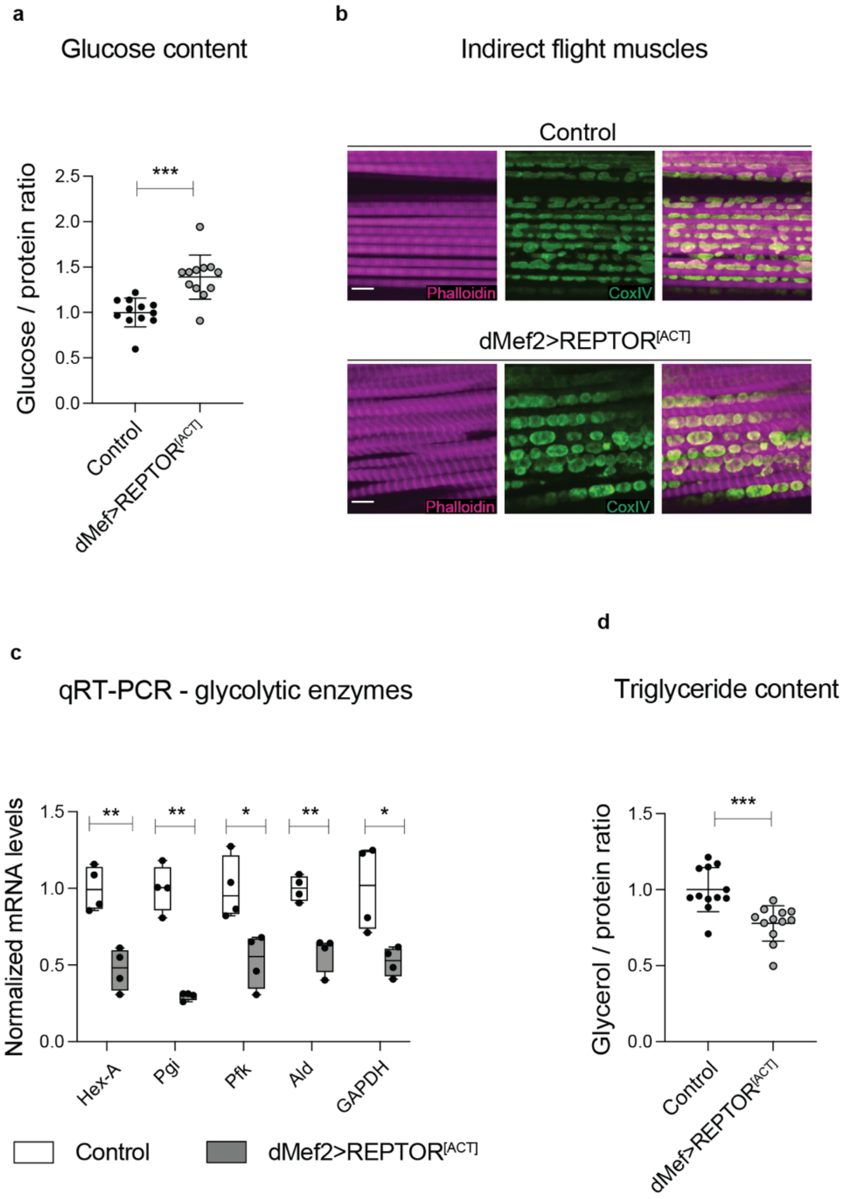

Supplementary Figure 7: *REPTOR[ACT]* induction in muscle modulates glucose metabolism and promotes myofiber degradation. **a** Glucose content in thoraces (p=0.0002***). N=12 biologically independent samples, N=8 thoraces per sample. Results were reproduced in three independent experiments. **b** Immunostaining of flight muscles in hemi-thoraces upon 8 days of *REPTOR[ACT]* overexpression. Myofibrils were labelled with phalloidin (magenta) and mitochondria with CoxIV (green). **c** mRNA levels of the first five enzymes in the glycolytic pathway *Hexokinase-a* (*hex-a), Phosphoglucose isomerase (pgi)*, *Phosphofructokinase* (*pfk*) *Aldolase (ald)* and *GAPDH* in thoraces upon *REPTOR[ACT]* induction in muscle (*hex-a* p=0.0018**, *pgi* p=0.0023**, *pfk* p=0.013*, *ald* p=0.001**, *GAPDH* p=0.035*). N=4 biologically independent samples, N=5-10 thoraces per sample. **d** Triglyceride content in thoraces (p=0.0005***). N=12 biologically independent samples, N=8 thoraces per sample. Triglyceride content was calculated from the same samples as in (**a**). Results were reproduced in three independent experiments.

Data shows mean with ± SD (**a, d**) or boxplots (median and quartiles) with whiskers (minimum to maximum) (**c**). Values were normalized to the mean of control samples (**a, c, d**). Statistical analysis was done by using two-tailed *t*-test with Welch's correction (**a, c, d**). Scale bar is 5 µm in **b.** Source data are provided as a Source Data file.

Supplementary Figure 8

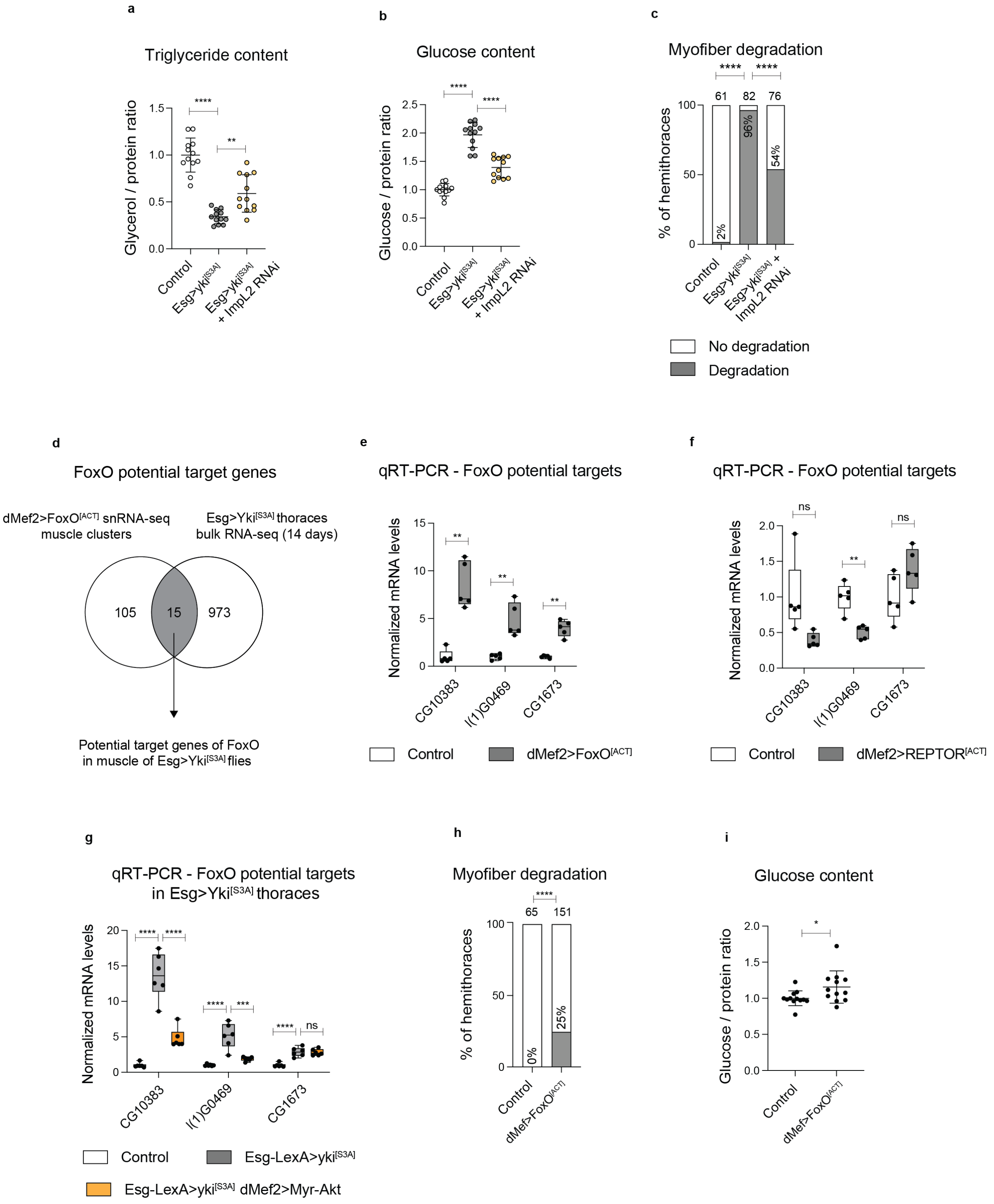

Supplementary Figure 8: *FoxO$^{[ACT]}$* overexpression in muscle does not phenocopy the overexpression of *REPTOR$^{[ACT]}$*. a, b Triglyceride (**a**) and glucose (**b**) content in thoraces (Triglycerides: Ctrl vs *Yki* p<0.0001****, *Yki* vs *Yki* + *ImpL2 RNAi* p=0.0013**. Glucose: Ctrl vs *Yki* p<0.0001****, *Yki* vs *Yki* + *ImpL2 RNAi* <0.0001****). N=12 biologically independent samples, N=8 thoraces per sample. Results were reproduced in three independent experiments. **c** Percentage of hemi-thoraces showing myofiber degradation (p<0.0001****). Total number of hemi-thoraces scored is shown for each genotype. Samples were analyzed after 8 days (**a**), 14 days (**b**) and 20 days (**c**) after tumor induction. **d** Schematics of the strategy to identify specific target genes of FoxO in muscle (see Fig. 3 and Methods). From 15 potential target genes, only 3 candidates have FoxO DNA binding sites and are upregulated in more than one muscle cluster (Supplementary Data 2). **e-g** mRNA levels of potential targets of FoxO in thoraces upon muscle-specific overexpression of *FoxO$^{[ACT]}$* (*CG10383* p=0.0016**, *l(1)G0649* p=0.0076**, *CG1673* p=0.0012**) (**e**), of *REPTOR$^{[ACT]}$* (*CG10383* p=0.06 ns, *l(1)G0649* p=0.0027**, *CG1673* p=0.09 ns) (**f**), or elevation of insulin signaling (*Myr-AKT*) in muscle of *Esg>yki$^{[S3A]}$* flies for 12 days (*CG10383:* Ctrl vs *Yki* p<0.0001****, *Yki* vs *Yki* + *Myr-AKT* p<0.0001****. *l(1)G0649:* Ctrl vs *Yki* p<0.0001****, *Yki* vs *Yki* + *Myr-AKT* p=0.0001***. *CG1673:* Ctrl vs *Yki* p<0.0001****, *Yki* vs *Yki* + *Myr-AKT* p=0.97 ns) (**g**). N=5-6 biologically independent samples, N=5-10 thoraces per sample. **h** Percentage of hemi-thoraces showing myofiber degradation. Total number of hemi-thoraces scored for each genotype is shown. **i** Glucose content in thoraces (p=0.0444*). N=12 biologically independent samples, N=8 thoraces per sample. Results were reproduced in three independent experiments. *FoxO$^{[ACT]}$* was induced for 20 days (**h, i**).

Data shows mean with ± SD (**a, b, i**) or boxplots (median and quartiles) with whiskers (minimum to maximum) (**e-g**). Values were normalized to the mean of control samples (**a, b, e-g, i**). Statistical analysis was done by using one-way ANOVA with Sidak correction test for multiple comparisons (**a, b, g**), or two-tailed *t*-test with Welch's correction (**e, f, i**), or two-tailed Fisher's exact test with a confidence interval of 95% for pairwise comparisons between two groups (**c, h**). Source data are provided as a Source Data file.

Supplementary Figure 9

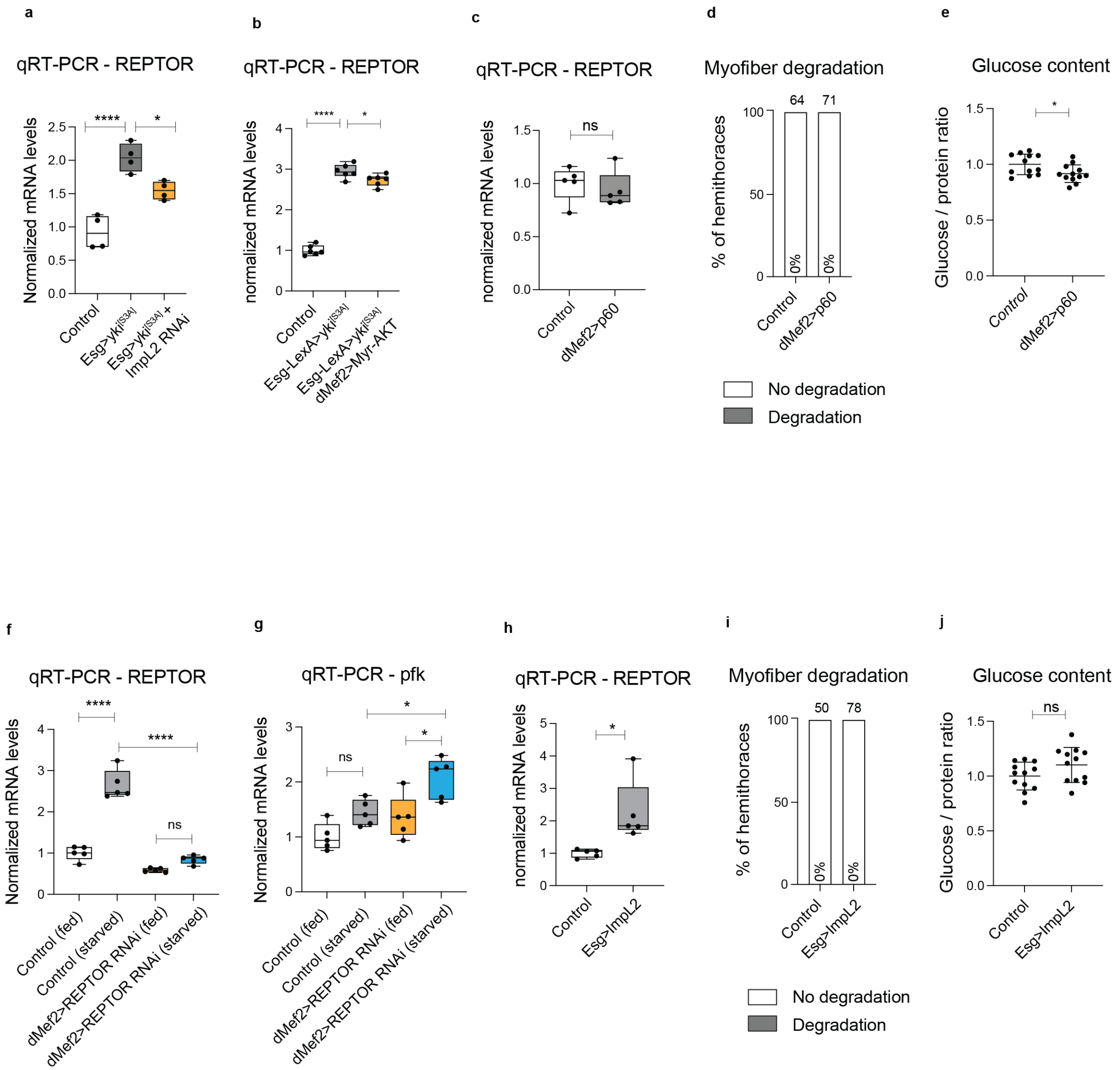

Supplementary Figure 9: Systemic reduction of insulin signaling in wildtype flies induces *REPTOR* expression. a-c mRNA levels of *REPTOR* in thoraces: upon *ImpL2* knockdown in gut *yki*-tumors after 20 days (Ctrl vs *Yki* p<0.0001****, *Yki* vs *Yki* + *ImpL2 RNAi* p=0.0169*) (**a**), with elevated insulin signaling (*Myr-AKT*) in muscle of *Esg>yki*$^{[S3A]}$ flies after 12 days (Ctrl vs *Yki* p<0.0001****, *Yki* vs *Yki* + *Myr-AKT* p=0.0399*) (**b**), with muscle-specific reduction of insulin signaling (*p60)* in wildtype flies (p=0.58 ns) (**c**). N=4-6 biologically independent samples, N=5-10 thoraces per sample. **d** Percentage of hemi-thoraces showing myofiber degradation. Total number of hemi-thoraces scored for each genotype is shown. **e** Glucose content in thoraces p=0.0259*). N=12 biologically independent samples, N=8 thoraces per sample. Results were reproduced in three independent experiments. *p60* was overexpressed in muscle for 20 days (**d, e**). **f, g** mRNA levels of *REPTOR* (**f**) or *pfk* (**g**) in thoraces under nutritional restriction (*REPTOR:* Ctrl fed vs Ctrl starved p<0.0001****, Ctrl starved vs REPTOR RNAi starved p<0.0001****, REPTOR RNAi fed vs REPTOR RNAi starved p=0.34 ns. *Pfk*: Ctrl fed vs Ctrl starved p=0.25 ns, Ctrl starved vs REPTOR RNAi starved p=0.0383*, REPTOR RNAi fed vs REPTOR RNAi starved p=0.017*). **h** mRNA levels of *REPTOR* in thoraces (p=0.0376*). N=5 biologically independent samples, N=5-10 thoraces per sample (**f-h**). **i** Percentage of hemi-thoraces showing myofiber degradation. Total number of hemi-thoraces scored for each genotype is shown. **j** Glucose content in thoraces (p=0.1 ns). N=12 biologically independent samples, N=8 thoraces per sample. Results were reproduced in three independent experiments. *Impl2* was overexpressed in wildtype guts for 20 days (**h, i**) or 14 days (**j**).

Data shows mean with ± SD (**e, j**) or boxplots (median and quartiles) with whiskers (minimum to maximum) (**a-c, f-h**). Values were normalized to the mean of control samples (**a-c, e-h, j**). Statistical analysis was done by using one-way ANOVA with Sidak correction test for multiple comparisons (**a, b, f, g**), two-tailed *t*-test with Welch's correction (**c, e, h, j**), or two-tailed Fisher's exact test with a confidence interval of 95% for pairwise comparisons between two groups (**d, i**). Source data are provided as a Source Data file.

Supplementary Figure 10

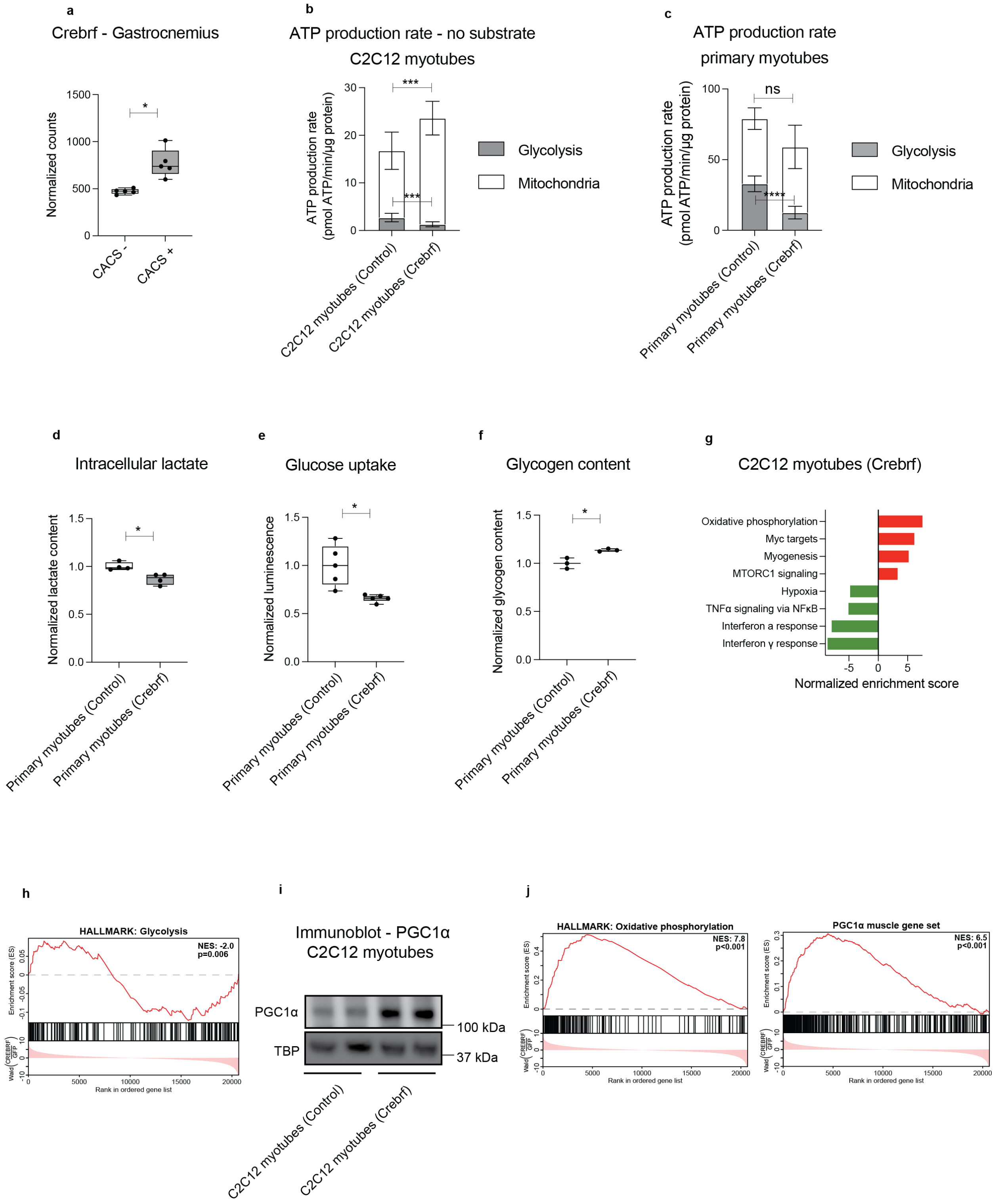

Supplementary Figure 10: Characterization of the gene expression program driven by CREBRF in mammalian myotubes. a *Crebrf* mRNA levels assessed by RNA-seq in mouse quadriceps of mice harboring non-small cell lung cancer that did (CACS +) or did not (CACS -) exhibit cachexia. Data adapted from a published dataset[1] (p=0.014*). N=5 biologically independent samples. **b** ATP production rate contributed by glycolysis and mitochondrial respiration in C2C12 myotubes upon adenoviral expression of *Crebrf*. No glucose, pyruvate, or glutamine were present in the media (Glycolysis p=0.0006***, Mitochondria p=0.0001***). **c** ATP production rate contributed by glycolysis and mitochondrial respiration in myotubes derived from primary cells upon adenoviral expression of *Crebrf* (Glycolysis p<0.0001****, Mitochondria p=0.94 ns). Values for glycolysis and mitochondrial respiration were calculated from N=10 biologically independent bioanalyzer wells for each condition (**b, c**). **d-f** lactate (p=0.011*) (**d**), glucose uptake (p=0.0226*) (**e**) and glycogen content (p=0.0435*) (**f**) upon *Crebrf* expression in myotubes derived from primary myoblasts. N=3-5 biologically independent samples. **g** Top four HALLMARK gene sets enriched amongst genes up- or down-regulated upon *Crebrf* expression in C2C12 myotubes, as determined by RNA-seq and Gene Set Enrichment Analysis (p<0.001 for each gene set). N=3 biologically independent samples. **h** Gene set enrichment plot assessing the HALLMARK glycolysis gene set in C2C12 myotubes expressing *Crebrf*. **i** Immunoblot of PGC1α protein in C2C12 myotubes expressing *Gfp* or *Crebrf*. TBP immunoblot of input. N=2 biologically independent samples. **j** Gene set enrichment plots assessing the expression of the PGC1α muscle gene set (Supplementary Data 1) or the HALLMARK oxidative phosphorylation gene set. Genes detected in RNA-seq were ranked based on Wald statistic, with the first ranked gene corresponding to the gene most significantly upregulated in *Crebrf* cells. Hash marks represent positions in the ranked list corresponding to members of a given gene set. Normalized enrichment score (NES) indicates whether these members are enriched toward the up-regulated end of this list (positive NES) or the downregulated end of this list (negative NES) as compared to chance expectation. Results were reproduced in two independent experiments (**b-f**).

Data shows mean with ± SD (**b, c**) or boxplots (median and quartiles) with whiskers (minimum to maximum) (**a, d-f**). Values were normalized to the average of control samples (**a, d-f**). Statistical analysis was done using two-tailed *t*-test with Welch's correction (**a, d-f**). Source data are provided as a Source Data file.

Supplementary Table 1 – qPCR primers used in this work

**qRT-PCR Primers - *Drosophila***	**Sequence 5'-3'**	**Source**
aTubulin-Fwd	CAACCAGATGGTCAAGTGCG	2
aTubulin-Rev	ACGTCCTTGGGCACAACATC	
Rp49-Fwd	ATCGGTTACGGATCGAACAA	3
Rp49-Rev	GACAATCTCCTTGCGCTTCT	
4E-BP-Fwd	TCCTGGAGGCACCAAACTTATC	4
4E-BP-Rev	GGAGCCACGGAGATTCTTCA	
REPTOR-Fwd	GCCGAGAGCTTCTCTTCAC	5
REPTOR-Rev	CGTCCTGCGAGAAGTCAAT	
ImpL2-Fwd	AAGAGCCGTGGACCTGGTA	6
ImpL2-Rev	TTGGTGAACTTGAGCCAGTCG	
Yki-Fwd	TCGCCGATCAAGTCCAACAA	This work. Kindly provided by Raghuvir Viswanatha
Yki-Rev	CGTGAAGAAGGAGTTGGGCA	
Hex-A-Fwd	CTGCTTCTAACGGACGAACAG	6
Hex-A-Rev	GCCTTGGGATGTGTATCCTTGG	
Hex-C-Fwd	GCGGAGGTGCGAGAACTTAT	6
Hex-C-Rev	CCACTTCCAGGCAAAAGCGA	
Pfk-Fwd	CGAGCCTGTGTCCGTATGG	6
Pfk-Rev	AGTTGGCTTCCTGGATGCAG	
Pyk-Fwd	GCAGGAGCTGATACCCAACTG	6
Pyk-Rev	CGTGCGATCCGTGAGAGAA	
Pgi-Fwd	ACTGTCAATCTGTCTGTCCA	7
Pgi-Rev	GATAACAGGAGCATTCTTCTCG	
Ald-Fwd	GCCCAGAAAATCGTTGCCC	6
Ald-Rev	GGGTCAGTGCTGAACAACAG	
GAPDH-Fwd	CCAATGTCTCCGTTGTGGA	3
GAPDH-Rev	TCGGTGTAGCCCAGGATT	
CG42390-Fwd	GCTCAGTACAACGAACTAACCG	This work. FlyPrimerBank: PP20233
CG42390-Rev	CAGTATGCGTATTTGCTCCTCC	
Gadd45-Fwd	ACATGCACGAGGTACTGCTG	This work. FlyPrimerBank: PP42383
Gadd45-Rev	CGCAGTAGTCGACTAGCTGG	
RhoGAP68F-Fwd	CCAACTCAGGGAGCAGTCTG	This work
RhoGAP68F-Rev	GGATTTTTCGGGAAAGCGGG	

CG10383-Fwd	TCTGCTCAATATCGCCCAAAC	This work. FlyPrimerBank: PP30860
CG10383-Rev	CCCAGTCCTTCAAGTGATCCA	
CG1673-Fwd	GGCGGACTTCGAGATCACC	This work. FlyPrimerBank: PP13646
CG1673-Rev	GACCCAGATTCTTGTCATGCTTA	
l(1)G0469-Fwd	GCATCAGAATCCCACAACCG	This work. FlyPrimerBank: PP13032
l(1)G0469-Rev	GGATCTCGACCACGAAACCG	

**qRT-PCR Primers - mouse**	**Sequence 5'-3'**	**Source**
Crebrf-Fwd	AGCGTAAGCGGAATGGACC	8
Crebrf-Rev	CAGGACATCTGTGAAAGTCTCC	
Tbp-Fwd	GAAGCTGCGGTACAATTCCAG	8
Tbp-Rev	CCCCTTGTACCCTTCACCAAT	
Rplp0-Fwd	AGATTCGGGATATGCTGTTGGC	8
Rplp0-Rev	TCGGGTCCTAGACCAGTGTTC	

Source Data - Supplementary Figure 2a: Immunoblot of pAKT and total AKT in *Drosophila* thoraces

Biological replicate 1

anti-pAKT immunoblot in protein extracts of thoraces (Cell Signaling 4060 - 1:1000)

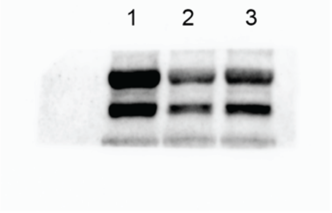

Lane 1 - Control
Lane 2 - Esg>Yki[S3A]
Lane 3 - Esg>Yki[S3A] + ImpL2 RNAi

Reblott with anti-AKT (Cell Signaling 9272 - 1:1000)

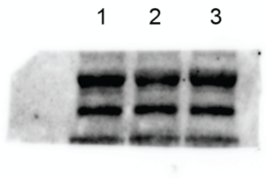

Lane 1 - Control
Lane 2 - Esg>Yki[S3A]
Lane 3 - Esg>Yki[S3A] + ImpL2 RNAi

anti-Tubulin immunoblot (Sigma T5168 - 1:20000)

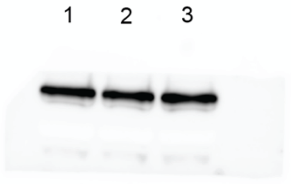

Lane 1 - Control
Lane 2 - Esg>Yki[S3A]
Lane 3 - Esg>Yki[S3A] + ImpL2 RNAi

	Densitometry of bands: Adj. Volume			
	pAKT	Total AKT	tubulin	pAKT/Total AKT ratio
Control	49603008	49130600	66125794	1
Esg>Yki[S3A]	14636352	38909855	59090619	0.372578069
Esg>Yki[S3A] + ImpL2 RNAi	22951584	41383532	65297302	0.549324747

Lower AKT band was used for analysis (approximately 60 kDa)
Tubulin is approximately 45 kDa

Loading control - Ponceau staining

1 2 3 4 5 6 7 8 9

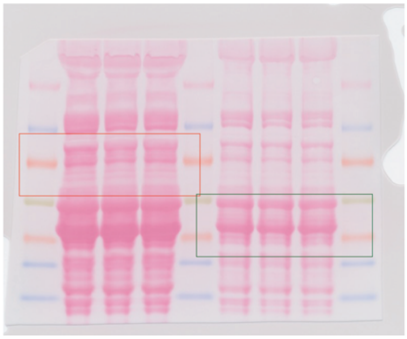

Lane 1 - Spectra Multicolor Broad Range Protein Ladder (Pierce #26634)
Lane 2 - Control (30-40 ug of protein)
Lane 3 - Esg>Yki[S3A] (30-40 ug of protein)
Lane 4 - Esg>Yki[S3A] + ImpL2 RNAi (30-40 ug of protein)
Lane 5 - Spectra Multicolor Broad Range Protein Ladder (Pierce #26634)
Lane 6 - Control (10-15 ug of protein)
Lane 7 - Esg>Yki[S3A] (10-15 ug of protein)
Lane 8 - Esg>Yki[S3A] + ImpL2 RNAi (10-15 ug of protein)
Lane 9 - Spectra Multicolor Broad Range Protein Ladder (Pierce #26634)

Membrane used for pAKT and total AKT immunoblot (red)
Membrane used for tubulin immunoblot (green)

Source Data - Supplementary Figure 2a: Immunoblot of pAKT and total AKT in *Drosophila* thoraces

Biological replicate 2

anti-pAKT immunoblot in protein extracts of thoraces (Cell Signaling 4060 - 1:1000)

1 2 3

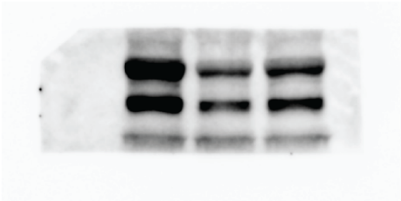

Lane 1 - Control
Lane 2 - Esg>Yki[S3A]
Lane 3 - Esg>Yki[S3A] + ImpL2 RNAi

Reblott with anti-AKT (Cell Signaling 9272 - 1:1000)

1 2 3

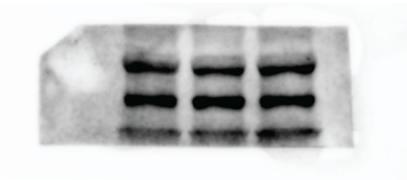

Lane 1 - Control
Lane 2 - Esg>Yki[S3A]
Lane 3 - Esg>Yki[S3A] + ImpL2 RNAi

anti-Tubulin immunoblot (Sigma T5168 - 1:20000)

1 2 3

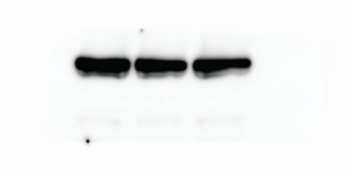

Lane 1 - Control
Lane 2 - Esg>Yki[S3A]
Lane 3 - Esg>Yki[S3A] + ImpL2 RNAi

	Densitometry of bands: Adj. Volume			
	pAKT	Total AKT	tubulin	pAKT/Total AKT ratio
Control	44773680	25398820	100729202	1
Esg>Yki[S3A]	12627132	37056712	84371115	0.193298546
Esg>Yki[S3A] + ImpL2 RNAi	18995050	30740130	78973314	0.35053024

Lower AKT band was used for analysis (approximately 60 kDa)
Tubulin is approximately 45 kDa

Loading control - Ponceau staining

1 2 3 4 5 6 7 8 9

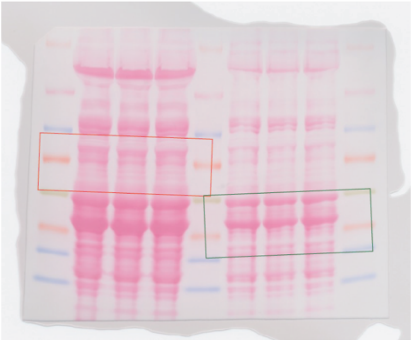

Lane 1 - Spectra Multicolor Broad Range Protein Ladder (Pierce #26634)
Lane 2 - Control (30-40 ug of protein)
Lane 3 - Esg>Yki[S3A] (30-40 ug of protein)
Lane 4 - Esg>Yki[S3A] + ImpL2 RNAi (30-40 ug of protein)
Lane 5 - Spectra Multicolor Broad Range Protein Ladder (Pierce #26634)
Lane 6 - Control (10-15 ug of protein)
Lane 7 - Esg>Yki[S3A] (10-15 ug of protein)
Lane 8 - Esg>Yki[S3A] + ImpL2 RNAi (10-15 ug of protein)
Lane 9 - Spectra Multicolor Broad Range Protein Ladder (Pierce #26634)

Membrane used for pAKT and total AKT immunoblot (red)
Membrane used for tubulin immunoblot (green)

Source Data - Supplementary Figure 5e: Immunoblot of pS6K and total S6K in *Drosophila* thoraces

Biological replicate 1

anti-pS6K immunoblot in protein extracts from thoraces (Cell Signaling 9209 - 1:1000)

1 2 3

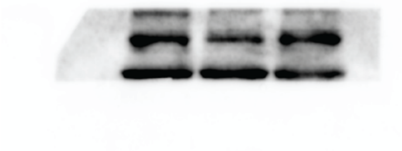

Lane 1 - Control
Lane 2 - Esg-LexA>Yki[S3A]-GFP
Lane 3 - Esg-LexA>Yki[S3A]-GFP + dMef2>Myr-AKT

Reblott with anti-S6K (PMID: 20444422 - 1:10000)

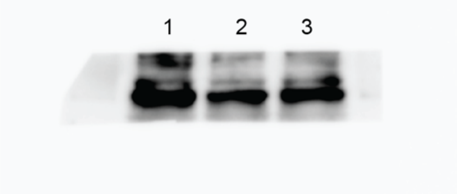

Lane 1 - Control
Lane 2 - Esg-LexA>Yki[S3A]-GFP
Lane 3 - Esg-LexA>Yki[S3A]-GFP + dMef2>Myr-AKT

anti-Tubulin immunoblot (Sigma T5168 - 1:20000)

1 2 3

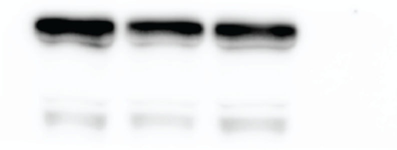

Lane 1 - Control
Lane 2 - Esg-LexA>Yki[S3A]-GFP
Lane 3 - Esg-LexA>Yki[S3A]-GFP + dMef2>Myr-AKT

	Densitometry of bands: Adj. Volume			
	**pS6K**	**total s6K**	**tubulin**	**pS6K/ Total S6K ratio**
Lane 1 - Control	53468976	56821408	78741663	1
Lane 2 - Esg-LexA>Yki[S3A]-GFP	34309315	32537916	55345108	1.120553091
Lane 3 - Esg-LexA>Yki[S3A]-GFP + dMef2>Myr-AKT	62329215	36764212	57805202	1.801675279

pS6K and total S6K bands are approximately 60 kDa
Tubulin is approximately 45 kDa

Loading control - Ponceau staining

1 2 3 4 5 6 7 8 9

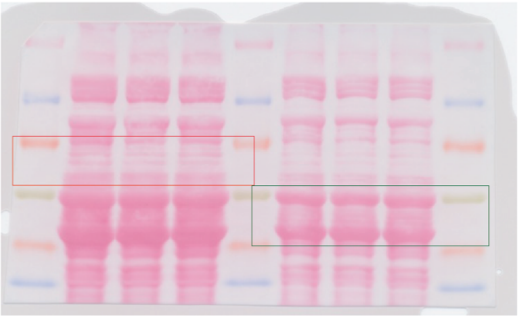

Lane 1 - Spectra Multicolor Broad Range Protein Ladder (Pierce #26634)
Lane 2 - Control (30-40 ug of protein)
Lane 3 - Esg-LexA>Yki[S3A]-GFP (30-40 ug of protein)
Lane 4 - Esg-LexA>Yki[S3A]-GFP + dMef2>Myr-AKT (30-40 ug of protein)
Lane 5 - Spectra Multicolor Broad Range Protein Ladder (Pierce #26634)
Lane 6 - Control (10-15 ug of protein)
Lane 7 - Esg-LexA>Yki[S3A]-GFP (10-15 ug of protein)
Lane 8 - Esg-LexA>Yki[S3A]-GFP + dMef2>Myr-AKT (10-15 ug of protein)
Lane 9 - Spectra Multicolor Broad Range Protein Ladder (Pierce #26634)

Membrane used for pS6K and total S6K immunoblot (red)
Membrane used for tubulin immunoblot (green)

Source Data - Supplementary Figure 5e: Immunoblot of pS6K and total S6K in *Drosophila* thoraces

Biological replicate 2

anti-pS6K immunoblot in protein extracts from thoraces (Cell Signaling 9209 - 1:1000)

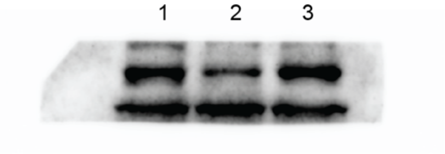

Lane 1 - Control
Lane 2 - Esg-LexA>Yki[S3A]-GFP
Lane 3 - Esg-LexA>Yki[S3A]-GFP + dMef2>Myr-AKT

Reblott with anti-S6K (PMID: 20444422 - 1:10000)

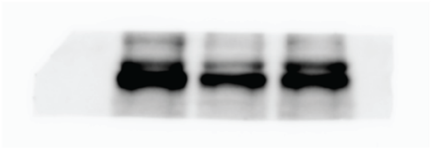

Lane 1 - Control
Lane 2 - Esg-LexA>Yki[S3A]-GFP
Lane 3 - Esg-LexA>Yki[S3A]-GFP + dMef2>Myr-AKT

anti-Tubulin immunoblot (Sigma T5168 - 1:20000)

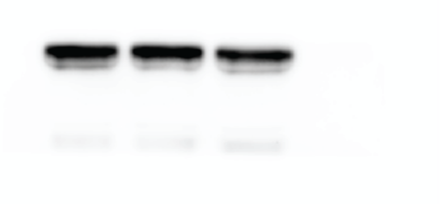

Lane 1 - Control
Lane 2 - Esg-LexA>Yki[S3A]-GFP
Lane 3 - Esg-LexA>Yki[S3A]-GFP + dMef2>Myr-AKT

	Densitometry of bands: Adj. Volume			
	pS6K	total s6K	tubulin	pS6K/ Total S6K ratio
Lane 1 - Control	43431084	59952480	68282832	1
Lane 2 - Esg-LexA>Yki[S3A]-GFP	28543569	34120416	67504422	1.154783158
Lane 3 - Esg-LexA>Yki[S3A]-GFP + dMef2>Myr-AKT	54491905	39335040	57594249	1.912312564

pS6K and total S6K bands are approximately 60 kDa
Tubulin is approximately 45 kDa

Loading control - Ponceau staining

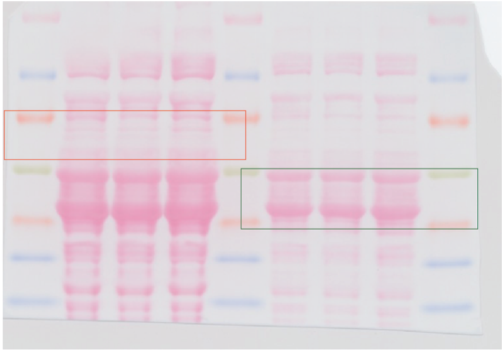

Lane 1 - Spectra Multicolor Broad Range Protein Ladder (Pierce #26634)
Lane 2 - Control (30-40 ug of protein)
Lane 3 - Esg-LexA>Yki[S3A]-GFP (30-40 ug of protein)
Lane 4 - Esg-LexA>Yki[S3A]-GFP + dMef2>Myr-AKT (30-40 ug of protein)
Lane 5 - Spectra Multicolor Broad Range Protein Ladder (Pierce #26634)
Lane 6 - Control (10-15 ug of protein)
Lane 7 - Esg-LexA>Yki[S3A]-GFP (10-15 ug of protein)
Lane 8 - Esg-LexA>Yki[S3A]-GFP + dMef2>Myr-AKT (10-15 ug of protein)
Lane 9 - Spectra Multicolor Broad Range Protein Ladder (Pierce #26634)

Membrane used for pS6K and total S6K immunoblot (red)
Membrane used for tubulin immunoblot (green)

Source Data - Supplementary Figure 10i: Immunoblot of Pgc1α in C2C12 myotubes

anti-Pgc1α immunoblot of protein extracts from C2C12 myotubes (EMD Millipore ST1202 - 1:1000)

1 2 3 4

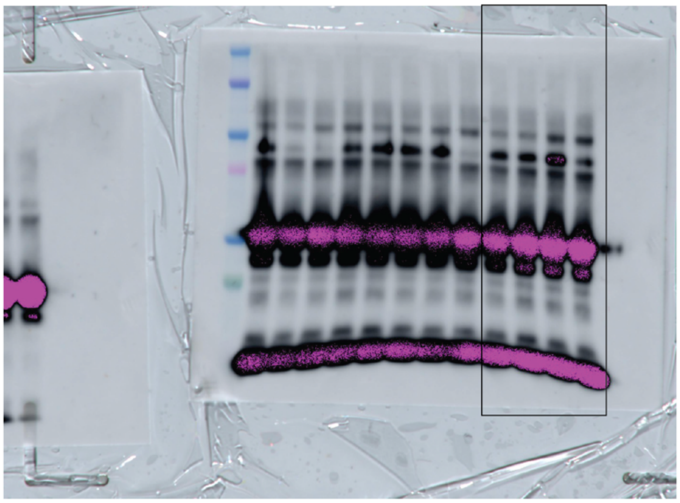

Pgc1α band is approximately 100 kDa

anti-Tbp immunoblot of protein extracts from C2C12 myotubes (Cell Signaling 44059 - 1:1000)

1 2 3 4

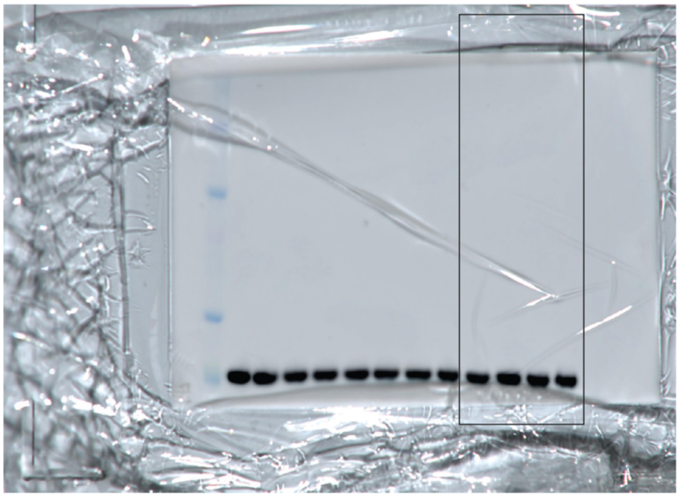

Tbp band is approximately 40 kDa

Lane 1 - Biological replicate 1 C2C12 myotubes (Control)
Lane 2 - Biological replicate 2 C2C12 myotubes (Control)
Lane 3 - Biological replicate 1 C2C12 myotubes (Crebrf overexpression)
Lane 4 - Biological replicate 2 C2C12 myotubes (Crebrf overexpression)

Supplementary references

1 Queiroz, A. L. *et al.* Blocking ActRIIB and restoring appetite reverses cachexia and improves survival in mice with lung cancer. *Nat Commun* **13**, 4633, doi:10.1038/s41467-022-32135-0 (2022).

2 Zirin, J. *et al.* Interspecies analysis of MYC targets identifies tRNA synthetases as mediators of growth and survival in MYC-overexpressing cells. *Proc Natl Acad Sci U S A* **116**, 14614-14619, doi:10.1073/pnas.1821863116 (2019).

3 Ewen-Campen, B. *et al.* Optimized strategy for in vivo Cas9-activation in Drosophila. *Proc Natl Acad Sci U S A* **114**, 9409-9414, doi:10.1073/pnas.1707635114 (2017).

4 Demontis, F. & Perrimon, N. FOXO/4E-BP signaling in Drosophila muscles regulates organism-wide proteostasis during aging. *Cell* **143**, 813-825, doi:10.1016/j.cell.2010.10.007 (2010).

5 Tiebe, M. *et al.* REPTOR and REPTOR-BP Regulate Organismal Metabolism and Transcription Downstream of TORC1. *Dev Cell* **33**, 272-284, doi:10.1016/j.devcel.2015.03.013 (2015).

6 Kwon, Y. *et al.* Systemic organ wasting induced by localized expression of the secreted insulin/IGF antagonist ImpL2. *Dev Cell* **33**, 36-46, doi:10.1016/j.devcel.2015.02.012 (2015).

7 Krejcova, G. *et al.* Drosophila macrophages switch to aerobic glycolysis to mount effective antibacterial defense. *Elife* **8**, doi:10.7554/eLife.50414 (2019).

8 Wang, X., Spandidos, A., Wang, H. & Seed, B. PrimerBank: a PCR primer database for quantitative gene expression analysis, 2012 update. *Nucleic Acids Res* **40**, D1144-1149, doi:10.1093/nar/gkr1013 (2012).
