# Supplementary material for: REPTOR and CREBRF encode key regulators of muscle energy metabolism
Source: Nat Commun. 2023 Aug 15;14:4943. doi: 10.1038/s41467-023-40595-1 (PMC10427696; doi:10.1038/s41467-023-40595-1)
Supplement: Supplementary file 3 — Description of Additional Supplementary Files [file 41467_2023_40595_MOESM3_ESM.pdf]

Description of Additional Supplementary Files Document

Supplementary Dataset 1:

Analysis of the bulk RNA-seq datasets and list of genes from the ‘PGC1α muscle gene set.’

Supplementary Dataset 2:

Analysis of the snRNA-seq dataset

Supplementary Dataset 3:

List of the Drosophila genotypes for each figure

Supplementary Dataset 4:

Targeted Mass Spectrometry values for all metabolites quantified in this work and respective ID database.
