# Supplementary material for: REPTOR and CREBRF encode key regulators of muscle energy metabolism
Source: Nat Commun. 2023 Aug 15;14:4943. doi: 10.1038/s41467-023-40595-1 (PMC10427696; doi:10.1038/s41467-023-40595-1)
Supplement: Supplementary file 8 — Reporting Summary [file 41467_2023_40595_MOESM8_ESM.pdf]

nature portfolio

Corresponding author(s): Pedro Saavedra and Norbert Perrimon

Last updated by author(s): Jul 24, 2023

Reporting Summary

Nature Portfolio wishes to improve the reproducibility of the work that we publish. This form provides structure for consistency and transparency in reporting. For further information on Nature Portfolio policies, see our Editorial Policies and the Editorial Policy Checklist.

Statistics

For all statistical analyses, confirm that the following items are present in the figure legend, table legend, main text, or Methods section.

n/a	Confirmed	
☐	☒	The exact sample size (n) for each experimental group/condition, given as a discrete number and unit of measurement
☐	☒	A statement on whether measurements were taken from distinct samples or whether the same sample was measured repeatedly
☐	☒	The statistical test(s) used AND whether they are one- or two-sided<br>*Only common tests should be described solely by name; describe more complex techniques in the Methods section.*
☒	☐	A description of all covariates tested
☐	☒	A description of any assumptions or corrections, such as tests of normality and adjustment for multiple comparisons
☐	☒	A full description of the statistical parameters including central tendency (e.g. means) or other basic estimates (e.g. regression coefficient) AND variation (e.g. standard deviation) or associated estimates of uncertainty (e.g. confidence intervals)
☐	☒	For null hypothesis testing, the test statistic (e.g. F, t, r) with confidence intervals, effect sizes, degrees of freedom and P value noted<br>*Give P values as exact values whenever suitable.*
☒	☐	For Bayesian analysis, information on the choice of priors and Markov chain Monte Carlo settings
☒	☐	For hierarchical and complex designs, identification of the appropriate level for tests and full reporting of outcomes
☒	☐	Estimates of effect sizes (e.g. Cohen's d, Pearson's r), indicating how they were calculated

Our web collection on statistics for biologists contains articles on many of the points above.

Software and code

Policy information about availability of computer code

Data collection	No software was used for data collection.
Data analysis	R (vs4.1.2) package: Harmony (vs0.10) and Seurat (vs4.3.0). DESeq2 (vs1.34.0), STAR (vs2.7.2b), PANGEA (vs1), DIOPT (vs9.2), Fiji (vs2.9.0), GraphPad Prism (vs9.5.1), Image Lab software (vs6.0.1), 10x Genomics Loupe Browser (vs6.4.1), 10x Genomics Cell Ranger (vs7.0.0). For the mammalian work, fastp (vs0.20.1); RNA STAR (vs2.7.8); DESeq2 (vs2.11.40.6); GSEA (vs4.0.1).

For manuscripts utilizing custom algorithms or software that are central to the research but not yet described in published literature, software must be made available to editors and reviewers. We strongly encourage code deposition in a community repository (e.g. GitHub). See the Nature Portfolio guidelines for submitting code & software for further information.

Data

Policy information about availability of data

All manuscripts must include a data availability statement. This statement should provide the following information, where applicable:

- Accession codes, unique identifiers, or web links for publicly available datasets
- A description of any restrictions on data availability
- For clinical datasets or third party data, please ensure that the statement adheres to our policy

The RNA-seq and snRNA-seq datasets generated in this work were deposited in the Gene Expression Omnibus (GEO) databases under the following accession codes:

GSE189214, GSE189218, GSE228034, GSE227038

Human research participants

Policy information about studies involving human research participants and Sex and Gender in Research.

Reporting on sex and gender	N/A
Population characteristics	N/A
Recruitment	N/A
Ethics oversight	N/A

Note that full information on the approval of the study protocol must also be provided in the manuscript.

Field-specific reporting

Please select the one below that is the best fit for your research. If you are not sure, read the appropriate sections before making your selection.

- [x] Life sciences
- [ ] Behavioural & social sciences
- [ ] Ecological, evolutionary & environmental sciences

For a reference copy of the document with all sections, see nature.com/documents/nr-reporting-summary-flat.pdf

Life sciences study design

All studies must disclose on these points even when the disclosure is negative.

Sample size	Sample size for quantification of protein, glucose and triglycerides in thoraces was based on the previous publications (PMID: 27572262, PMID: 27185732, PMID: 30554999). For ATP quantification, the sample size was based on a previous publication (PMID: 30872605). For qRT-PCR experiments, sample size was based on several publications. For mammalian experiments the number of biological replicates was based on similar experiments previously published (PMID: 28475869, PMID: 31235694). No statistical method was used to determine sample size.
Data exclusions	There were no data exclusions in any panel of results.
Replication	Quantification of protein, glucose and triglycerides and ATP content were replicated in all three independent experiments performed. Western blots results were replicated in three independent experiments, with the exception of the quantification of the pS6K/S6K ratio in Figure 2b that was replicated in two independent experiments that had comparable levels of total S6K across samples. Immunostainings showed similar results in five different animals analyzed. Results with mammalian samples were reproduced in the two independent experiments performed.
Randomization	Randomization was not applicable in this study. Genotypes were separated and grouped by genetic background.
Blinding	No blinding was done. The investigators set up the experiments, collected and analyzed the data making it impossible to perform blind analysis.

Reporting for specific materials, systems and methods

We require information from authors about some types of materials, experimental systems and methods used in many studies. Here, indicate whether each material, system or method listed is relevant to your study. If you are not sure if a list item applies to your research, read the appropriate section before selecting a response.

Materials & experimental systems

n/a	Involved in the study	
[ ]	[x]	Antibodies
[ ]	[x]	Eukaryotic cell lines
[x]	[ ]	Palaeontology and archaeology
[ ]	[x]	Animals and other organisms
[x]	[ ]	Clinical data
[x]	[ ]	Dual use research of concern

Methods

n/a	Involved in the study	
[x]	[ ]	ChIP-seq
[x]	[ ]	Flow cytometry
[x]	[ ]	MRI-based neuroimaging

Antibodies

Antibodies used	For immunostaining of Drosophila samples, primary antibodies used were mouse anti-CoxIV (1:200-1:500, Abcam – 33985), chicken anti-GFP (1:2000, Aves Labs – GFP-1020), mouse anti-bPS1 (1:50, DSHB – CF.6G11), mouse anti-dlg1 (1:50, DSHB – 4F3) and rabbit anti-amphiphysin (Dr Andrew Zelhof, PMID: 11711432 - #9906 - 1:200). Secondary antibodies used were anti-mouse Alexa Fluor 488 (1:500, Thermo Fisher Scientific – A-21202), anti-chicken 488 (1:500, Jackson ImmunoResearch – 703-545-155) or Alexa Fluor 647 (1:500, Thermo Fisher Scientific – A-21244).<br><br>For immunoblots of Drosophila samples, primary antibodies used were rabbit anti-pAKT (1:1000, Cell Signaling – 4060), rabbit anti-Akt (1:1000, Cell Signaling – 9272), rabbit anti-pS6K (1:1000, Cell Signaling – 9209), guinea pig anti-S6K (Dr Aurelio Teleman, PMID: 20444422 - 1:10000), mouse anti-tubulin (1:10000, Sigma – T5168), guinea pig anti-REPTOR (Dr Aurelio Teleman, PMID: 25920570 - 1:1000). Secondary antibodies used were anti-mouse HRP (1:10000, Amersham – NXA931), anti-rabbit HRP (1:10000, Amersham – NA934) and anti-guinea pig HRP (1:10000, Jackson ImmunoResearch – 106-035-003)<br><br>For immunoblots of mouse samples, primary antibodies used were rabbit anti-TBP (1:1000, Cell Signaling – 44059) and mouse anti-PGC1α (1:1000, EMD Millipore – ST1202). Secondary antibodies used were anti-rabbit HRP (Promega – W4011) or anti-mouse HRP (Promega – W4021)
Validation	Validation of commercially available antibodies:<br>anti-CoxIV (Abcam – 33985): https://www.abcam.com/products/primary-antibodies/cox-iv-antibody-mabcam33985-mitochondrial-marker-ab33985.html<br>anti-GFP (Aves Labs – GFP-1020): https://www.aveslabs.com/products/anti-green-fluorescent-protein-antibody-gfp<br>anti-bPS1 (DSHB – CF.6G11): https://dshb.biology.uiowa.edu/CF-6G11<br>anti-dlg1 (DSHB – 4F3): https://dshb.biology.uiowa.edu/4F3-anti-discs-large<br>anti-pAKT (Cell Signaling – 4060): https://www.cellsignal.com/products/primary-antibodies/phospho-akt-ser473-d9e-xp-rabbit-mab/4060<br>anti-Akt (Cell Signaling – 9272): https://www.cellsignal.com/products/primary-antibodies/akt-antibody/9272<br>anti-pS6K (Cell Signaling – 9209): https://www.cellsignal.com/products/primary-antibodies/phospho-drosophila-p70-s6-kinase-thr398-antibody/9209<br>anti-tubulin (Sigma – T5168): https://www.sigmaaldrich.com/US/en/product/sigma/t5168<br>anti-TBP (Cell Signaling – 44059): https://www.cellsignal.com/products/primary-antibodies/tbp-d5c9h-xp-rabbit-mab/44059<br>anti-PGC1α (EMD Millipore – ST1202): https://www.emdmillipore.com/US/en/product/Anti-PGC-1-Mouse-mAb-4C1.3,EMD_BIO-ST1202<br><br>For non-commercial antibodies the information is in the following relevant publications.<br>anti-amphiphysin, PMID: 11711432<br>anti-REPTOR, PMID: 25920570<br>anti-S6K, PMID: 20444422

Eukaryotic cell lines

Policy information about cell lines and Sex and Gender in Research

Cell line source(s)	C2C12 mouse myoblasts line obtained from ATCC (CRL-1772). HEK293A were obtained from Thermo Fisher (R70507).
Authentication	Cells obtained from ATCC and Thermo Fisher were not authenticated.
Mycoplasma contamination	Not tested for mycoplasma
Commonly misidentified lines<br>(See ICLAC register)	None used in this study.

Animals and other research organisms

Policy information about studies involving animals; ARRIVE guidelines recommended for reporting animal research, and Sex and Gender in Research

Laboratory animals	The strains of Drosophila melanogaster lines used in this work and their origin are listed: Bloomington Drosophila Stock Center (BDSC): esg.LexA::GAD (P{ST.lexA::HG}SJH-1, 66632). UAS-p60 (P{UAS-Pi3K21B.HA}2, 25899). UAS-Myr-Akt (P{UAS-myr-Akt.ΔPH}3, 80935). tub-GAL80[TS] (P{tubP-GAL80[ts]}10, 7108. P{tubP-GAL80ts}7, 7018); UAS-CPT1 RNAi (P{y[+t7.7] v[+t1.8]=TRiP.HMS00040} attP2, 34066). National Institute of Genetics Stock Center (NIG): UAS-ImpL2-RNAi (15009R-3). UAS-REPTOR-RNAi (13624R-3). Vienna Drosophila Research Center (VDRC): Act88f::GFP (PBac{fTRG10028.sfGFP-FT}, v318362). Laboratory stocks: w[1118]. tub-GAL80[ts], dmef2-GAL4. tub-GAL80[ts]; lpp-GAL4. esg-GAL4, UAS-GFP, tub-GAL80[ts] (PMID: 16340959). UAS-yki[S3A] (PMID: 19330023). UAS-s.ImpL2 (PMID: 18412985). UAS-Tsc1/Tsc2 (PMID: 11348591). UAS-Transtimer (PMID: 31140975). The stock UAS-Empty[VK33] (3rd chromosome) was a gift from Hugo Bellen laboratory. The stocks UAS-REPTOR[ACT] (PMID: 25920570) and UAS.PRAS40 (PMID: 22264732) were a gift from Aurelio Teleman. The stocks UAS-HA-FoxO[ACT], UAS-REPTOR[WT], LexAop-nls-sfGFP and LexAop-yki [S3A]-sfGFP were made in this work. A list of the genotypes used for each Figure is presented in Supplementary Data 3.<br><br>The age of the flies was precisely defined according to the nature of the experiment: for gut yki-tumors, eclosed male flies of the

	right genotype were collected every 24-48 hours and then incubated at 29°C (GAL4) for 2, 8 , 14 or 20 days, or 27°C (LexAop) for 12, 16 or 19 days, to induce yki[S3A] expression in the gut. For muscle-specific expression (dMef2-GAL4), new adult males were collected every 48-72 hours, kept at 18°C for an additional 4-5 days and then incubated at 29°C to induce gene expression for (4, 8 or 20 days).<br><br>Regarding the mammalian work, Mouse C57BL/6J were used and all animals were 8-12 weeks old.
Wild animals	No wild animals were collected or used in this study
Reporting on sex	All animals used in this study were males. Primary myoblasts were isolated from female C57BL/6J mice.
Field-collected samples	No field-collected samples were used in this work.
Ethics oversight	Protocols were approved by Animal Care and Use Committee of the Beth Israel Deaconess Medical Center.

Note that full information on the approval of the study protocol must also be provided in the manuscript.
